# Variability in Costs across Hospital Wards. A Study of Chinese Hospitals

**DOI:** 10.1371/journal.pone.0097874

**Published:** 2014-05-29

**Authors:** Taghreed Adam, David B. Evans, Bian Ying, Christopher J. L. Murray

**Affiliations:** 1 Alliance for Health Policy and Systems Research, World Health Organization, Geneva, Switzerland; 2 Health Systems Governance and Financing, World Health Organization, Geneva, Switzerland; 3 Institute of Chinese Medical Sciences, University of Macau, Macau, China; 4 Institute for Health Metrics and Evaluation, University of Washington, Seattle, Washington, United States of America; University of Washington, United States of America

## Abstract

**Introduction:**

Analysts estimating the costs or cost-effectiveness of health interventions requiring hospitalization often cut corners because they lack data and the costs of undertaking full step-down costing studies are high. They sometimes use the costs taken from a single hospital, sometimes use simple rules of thumb for allocating total hospital costs between general inpatient care and the outpatient department, and sometimes use the average cost of an inpatient bed-day instead of a ward-specific cost.

**Purpose:**

In this paper we explore for the first time the extent and the causes of variation in ward-specific costs across hospitals, using data from China. We then use the resulting model to show how ward-specific costs for hospitals outside the data set could be estimated using information on the determinants identified in the paper.

**Methodology:**

Ward-specific costs estimated using step-down costing methods from 41 hospitals in 12 provinces of China were used. We used seemingly unrelated regressions to identify the determinants of variability in the ratio of the costs of specific wards to that of the outpatient department, and explain how this can be used to generate ward-specific unit costs.

**Findings:**

Ward-specific unit costs varied considerably across hospitals, ranging from 1 to 24 times the unit cost in the outpatient department — average unit costs are not a good proxy for costs at specialty wards in general. The most important sources of variability were the number of staff and the level of capacity utilization.

**Practice Implications:**

More careful hospital costing studies are clearly needed. In the meantime, we have shown that in China it is possible to estimate ward-specific unit costs taking into account key determinants of variability in costs across wards. This might well be a better alternative than using simple rules of thumb or using estimates from a single study.

## Introduction

Information on hospital costs is an important input to the economic and financial analyses of many types of health interventions. Although there is strong evidence that unit costs of inpatient stays and outpatient visits vary substantially, even between similar hospitals within the same setting [1]–[3], analysts often have difficulty finding sufficient information on costs in their settings. Partly as a result, published costing and cost-effectiveness studies have used a number of shortcuts. They include basing their analyses on costs taken from one hospital, extrapolating costs estimated in one country to other countries, basing costs on a small study of a particular group of patients, or allocating total hospital costs between general inpatient care and the outpatient department according to an assumed ratio of the costs of an inpatient day to an outpatient visit [3]–[6]. Ratios of 4∶1 and 3∶1 can be found in the literature [3], [4].

Previous research has shown that simple rules of thumb do not provide an accurate basis for allocating total hospital costs between overall inpatient and outpatient care, largely due to variability across hospitals in such factors as hospital size or degree of specialization [3]. Adam and Evans, using a large, cross-country data set, then developed a way of allocating known hospital costs between inpatient and outpatient care, taking into account variability in these determinants [1]. It could be used to estimate the costs of an inpatient day in settings where data are unavailable and the costs of undertaking multi-hospital studies are prohibitive. This paper takes this work a step further, by exploring if the costs of an inpatient stay in specific wards differ to the costs of an overall inpatient bed-day across hospitals, and the reasons for this variation. We used seemingly unrelated regression analysis for this analysis since it offers an efficient approach to developing a system of equations where error terms are correlated. We also developed a way of estimating ward-specific costs across settings using information on total hospital costs, for specified values of the different determinants. A large data set from China was used to explore these questions.

## Methods

Previous studies had focused on the relationship between inpatient care in general and outpatient care, and had used multivariate regression analysis [1], [3]. Because our aim is to distinguish between the different types of speciality inpatient wards, a more efficient estimation method than the use of independent regression models for each ward is offered by seemingly unrelated regression techniques, building on applications found in a range of disciplines including economics, political science and epidemiology [7]–[12].

Our problem is to allocate the total hospital costs across the inpatient and outpatient wards, knowing that the sum of the ward-specific costs must equal total hospital costs (a characteristic of compositional data). Seemingly unrelated regressions are commonly used to estimate this type of relationship, with the model containing several equations and an additional identity [7], [13], [14]. This identity implies that the J dependent variables (in this case ward cost ratios) sum to a fixed known value (i.e. total hospital costs). The implicit adding-up condition causes the seemingly unrelated equations to be correlated, something that is necessary given that the different departments are within the same hospital. Subsequently, the error terms will inherit this correlation from the dependent variables, maximizing efficiency in the estimation.

Since the correlation between the residuals in the J categories or departments of a hospitals results in a singular covariance matrix [14], deleting one equation before the estimation procedure solves the problem and the 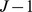 linear independent equations contain the entire statistical information required for estimation [15]. The choice of the equation that should be dropped does not affect the estimated parameters, so one arbitrary equation can be dropped and the remaining 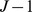 equations estimated [7], [14].

### Data

Forty-one hospitals from 12 provinces of China were included in the analysis (see Supplementary Information in Appendix S1 for the list of provinces). All the data were collected and analysed through the same study, using the same cost principles and data collection methods and analysis. The data were for the years 1997 and 2000 and unit costs were estimated using the standard step-down costing methodology [4], [16]. Cost categories included recurrent costs such as salaries, medicines, medical supplies, utilities and transport, and the annualized value of capital costs. All hospitals had inpatient wards for internal medicine; obstetrics and gynaecology; surgery; and paediatrics. All other inpatient wards were grouped into one category called “others”. The number of wards included in this category varied between one and seven depending on the hospital, and included ear nose and throat, dermatology, ophthalmology, physiotherapy and Chinese traditional medicine. The outpatient “ward” was the final category.

The database variables included total and unit cost per bed day and per admission for each ward. It also included hospital and ward-specific indicators such as level of hospital (primary, secondary or tertiary), number of beds, occupancy rate, average length of stay and utilization rates (e.g., number of bed-days used), as well as the year to which the cost data referred and the name of the province. In addition we included data on provincial GDP per capita obtained from official sources. Costs were converted to 2000 Chinese Yuan by means of the overall GDP deflator for China [17].

### Comparison of average unit cost ratios

**Figure 1:**
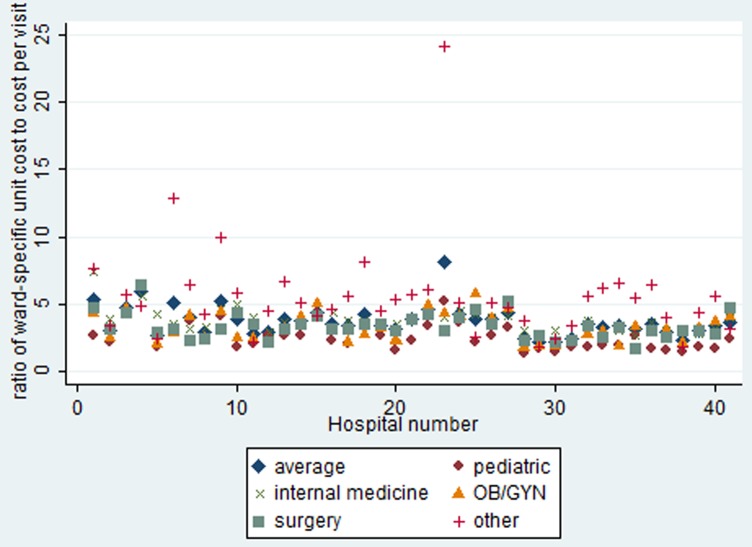
Comparison of the average and ward-specific unit cost per bed day to the unit cost per outpatient visit across the study hospitals. N = 41.

Before exploring the determinants of ward-specific cost ratios across hospitals, we explored the extent to which ward-specific unit costs differed. We computed the ratio of each ward-specific unit cost per inpatient day to the unit cost per outpatient visit. As an initial phase of analysis, differences in the ratios were compared using t tests, see Figure 1.

### Statistical model

In order to model compositional data with 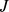 different hospital wards, we first define a vector of cost-proportions or the ratio of the ward-specific cost to the total cost, using the same notation as in [9] in an application to multiparty electoral data.
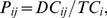
(1)Where 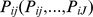 denotes the proportion of a particular ward's cost (DC) for each ward 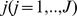 to total hospital cost (TC), for each hospital 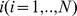. Six proportions were calculated for each hospital using the wards described earlier.

Second, a 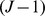 vector 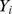 is generated by calculating the log ratios of each ward fraction relative to the dropped fraction j, in this case the outpatient ward, as follows [8]:
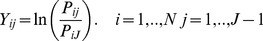
(2)where 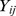 is the log of ward-specific cost over the outpatient ward cost; 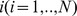 is the number of hospitals and 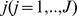 is the different wards *J* being the outpatient ward. The vector 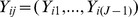 is assumed to be multivariate normal with mean *μ* and variance matrix Σ. The functional form is assumed to be linear in log scale.

### Model-fitting

The objective was to identify variables that would be likely to have a strong relationship to ward-specific costs. The variables for which data were available were occupancy rate, reflecting capacity utilization; the ratio of ward-specific bed days to total hospital bed-days as a measure of the proportion of overhead costs attributed to each ward; and number of staff per ward as a measure of ward size. In addition, various dummy variables were used to explore if hospitals from the different provinces in China behaved differently. Only the dummy for Henan province – the province where 40% of the data come from - proved significant, so it is the variable reported here. All the explanatory variables except the province dummy vary by ward. The final version of the model reported here included all variables in the natural log form. This transformation ensured that the distributions were approximately normal. It also provided the best fit of the various models tried.

A number of other explanatory variables were explored including: provincial GDP per capita, dummy variables for hospital type (primary, secondary, tertiary) and ward-specific variables such as the average length of stay, number of beds, bed days as a proportion of hospital beds and number of bed-days. Since they did not add any explanatory power they were not included in the reported model.

Given the specification described above, the system of equations for the log ratios may be written as follows:
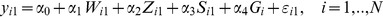
(3a)

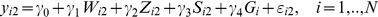
(3b)

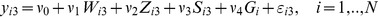
(3c)

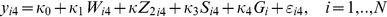
(3d)

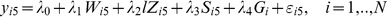
(3e)where 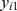 to 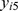 are the log-ratios as defined in Eq. (2); 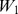 to 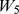 are ward-specific natural log of occupancy rate; 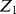 to 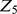 are ward-specific log ratios of ward bed-days over total hospital bed-days; 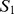 to 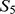 are ward-specific log of number of staff; 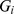 is a dummy variable for Henan province; and 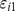 to 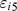 are the correlated residual terms. The asymptotically efficient, feasible generalized least-squares algorithm was computed using STATA 8 software [18], [19].

### Goodness of fit

Residual plots were used to check for normal distribution of the error terms, assumed by seemingly unrelated regression models. The percentage of variance explained by the explanatory variables was summarized by the adjusted R-squared. F statistics for testing the hypothesis of equal parameter vectors were calculated for each equation [18]. To test the hypothesis of independent equations - in which case it would not be necessary to use seemingly unrelated regressions - the correlation matrix of residuals and the Breusch-Pagan test for independent equations was performed [19].

### Predicted values and uncertainty intervals

We used the method described in Katz and King to estimate the ratios of ward-specific costs to total hospital cost and the uncertainty around these estimates [9]. This required several steps. First multiple simulation methods were used where, in each of 1000 iterations, a random draw was taken from a multivariate normal distribution around the estimators, with a mean vector consisting of the maximum likelihood estimates of the coefficients and the variance-covariance matrix derived from the regression results. The predicted values 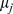 were then computed for the log-ratios P_1_ to P_5_, simulated 1000 times for each of the original values for the explanatory variables [20], [21]. From these simulations, the mean predicted value, standard deviation, and 95% confidence interval around the predicted values are computed. In this way, the analysis accounts for both fundamental and parameter uncertainty [20].

Second, the simulated predicted values 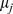 were back-transformed to natural units by multiplying their exponent by 1.12; the Duan smearing correction factor for the best-fit model. This is because one of the implicit assumptions of using log-transformed models is that the residuals in the transformed space are normally distributed. In this case, back-transforming gives the median and not the mean. To estimate the mean it is necessary to use a bias correction technique as describe by Duan 1983 [21], [22].

Third, the product from step two above was transformed into the initial proportions of ward costs to total hospital costs (see Eq. (1)) as follows:
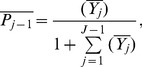
(4)with 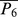 calculated as 1 –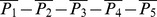.

Thus, in generating 1000 simulations of the fraction vector 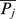, we can summarize the probability distribution of the predicted proportions of the six wards given values of the explanatory variables, and taking into account the uncertainty arising from using statistical models [20].

### Estimating ward-specific unit cost ratios

The estimated fractions are, however, not particularly useful by themselves. They simply show the ratio of the total costs of a specific inpatient ward to the outpatient ward. Of more interest to policy is the ratio of the cost of an inpatient day in a particular ward to the cost of an outpatient visit. Using the estimates from Eq. (4) and information on the number of bed days and outpatient visits from hospital records, the average ward-specific unit cost ratios - i.e. the cost of an inpatient day compared to the cost of an outpatient visit - can be derived as follows.
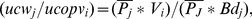
(5)where 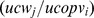 is the ratio of unit cost per bed day of the *jth* ward to the unit cost per outpatient visit in the outpatient ward, 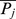 is the ratio of total cost of the *jth* ward to total hospital cost estimated from Eq. (4), 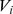 is total number of outpatient visits of hospital *i*, 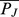 is the proportion that was omitted from the model (ratio of cost of outpatient ward to total cost) which corresponds to 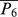 in Eq. (7) , and 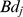 is the number of bed-days of the *jth* ward.

### Estimating ward-specific unit costs

From the ratios computed in Eq. (5), the unit cost of a particular ward can be calculated as:
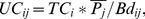
(6)where 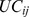 is the unit cost of the *jth* ward of hospital *i*, 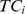 is total hospital cost, 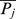 is the ratio of cost of the *jth* ward to total hospital cost (assuming an average ratio is used but the specific ratio for hospital *i* could be used instead) and 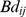 the number of bed days of the *jth* ward.

## Results

Figure 1 illustrates the substantial variability in unit cost ratios among the hospitals included in our study. In 90% of the cases, the cost of an inpatient bed-day lies between 1.5 and 9.5 times the cost of an outpatient visit, but 10% of observations obviously lie outside this range. At the extreme, the ratio was as high as 24 times the unit cost per outpatient visit in one hospital. The largest variability (coefficient of variation = 0.65) is, understandably, associated with the ward called “others” which includes a variety of different types of medical specialties. Variability is also relatively high for the paediatrics ward (coefficient of variation = 0.42) while it is lowest for the internal medicine ward (0.23).

On average, the unit cost per bed day in the paediatric ward was the lowest while it was highest in the ward for internal medicine, spanning almost 30% difference in unit costs. This can be explained by the significantly higher number of staff, on average, in internal medicine wards (133—see Table 1) compared with paediatric wards (27). No significant differences were observed in the average unit cost per bed day of the surgery and gynaecology wards across these hospitals. The costs of a stay in a specific type of ward can be as low as just over one-third (0.37 times) or as high as 6.5 times the average cost of an inpatient stay, depending on the hospital.

**Table 1:** Variable names, description and mean values.

Variable name	Description	Variable notation^1^	Mean in natural units	SD
Lnp**g**	**Dependent variable:** natural log-ratio of cost of *Ob/Gyn* to cost of outpatient wards		3.36	1.14
ln**g**_occupancy	Natural log of occupancy rate in *Ob/Gyn* ward	*W_1_*	0.87	0.42
ln**g**_bday_tbd	Natural log of ratio of bed days in *Ob/Gyn* ward to total bed days	*Z_1_*	0.08	0.06
ln**g**_staff	Natural log of number of staff in *Ob/Gyn* ward	*S_1_*	44.88	20.25
Henan	Dummy variable for Henan Province. Henan = 1	*Gi*		
Lnp**m**	**Dependent variable:** natural log-ratio of cost of *Internal Medicine* to cost of outpatient wards		3.82	0.89
ln**m**_occupancy	Natural log of occupancy rate in *Internal Medicine* ward	*W_2_*	0.87	0.35
ln**m**_bday_tbd	Natural log of ratio of bed days in *Internal Medicine* ward to total bed days	*Z_2_*	0.22	0.15
ln**m**_staff	Natural log of number of staff in *Internal Medicine* ward	*S_2_*	133.24	86.17
Henan	Dummy variable for Henan Province. Henan = 1	*Gi*		
Lnp**s**	**Dependent variable**: natural log-ratio of cost of *Surgery* to cost of outpatient wards		3.41	0.93
ln**s**_occupancy	Natural log of occupancy rate in *Surgery* ward	*W_3_*	0.93	0.47
ln**s**_bday_tbd	Natural log of ratio of bed days in *Surgery* ward to total bed days	*Z_3_*	0.22	0.16
ln**s**_staff	Natural log of number of staff in *Surgery* ward	*S_3_*	112.88	62.42
Henan	Dummy variable for Henan Province. Henan = 1	*Gi*		
Lnp**p**	**Dependent variable**: natural log-ratio of cost of *Paediatrics* to cost of outpatient wards		2.65	1.11
ln**p**_occupancy	Natural log of occupancy rate in *Paediatrics* ward	*W_4_*	0.76	0.38
ln**p**_bday_tbd	Natural log of ratio of bed days in *Paediatrics* ward to total bed days	*Z_4_*	0.04	0.03
ln**p**_staff	Natural log of number of staff in *Paediatrics* ward	*S_4_*	26.98	12.65
Henan	Dummy variable for Henan Province. Henan = 1	*Gi*		
Lnp**oth**	**Dependent variable**: natural log-ratio of cost of *other* wards to cost of outpatient ward		5.60	3.63
ln**oth**_occupancy	Natural log of occupancy rate in *other* wards	*W_5_*	0.73	0.41
ln**oth**_bday_tbd	Natural log of ratio of bed days in *other* wards to total bed days	*Z_5_*	0.13	0.11
ln**oth**_staff	Natural log of number of staff in *other* wards	*S_5_*	34.84	42.13
Henan	Dummy variable for Henan Province. Henan = 1	Gi		

1 See Equation 3(a) to 3(e).

As the first step in explaining these variations, we estimated the system of equations described in Eq. (3a–3e), showing the ratio of ward-specific total costs to the total cost of the outpatient department. Results are reported in Table 2. The model variables explained a large proportion of the variation in ward-specific ratios, the R-squared varying between 0.83 and 0.92. The F statistics for each equation are highly significant, varying between 112 and 372. Examination of the residual plots showed a normal distribution of the error terms. In addition, the null hypothesis of a zero correlation between residuals was rejected (Breusch-Pagan test, chi2(10) = 297, p<0.00001), which confirms the appropriateness of using seemingly unrelated regression for this analysis rather than estimating each equation separately.

**Table 2:** Results of the seemingly unrelated regression using iterated feasible generalized least squares estimates.

Equation	RMSE	R-squared	F-Statistic	P>F
lnp**g**	0.5229	0.8579	306.17	<0.0001
lnp**m**	0.4402	0.9095	238.45	<0.0001
lnp**s**	0.4384	0.9201	372.83	<0.0001
lnp**p**	0.5951	0.8942	112.91	<0.0001
lnp**oth**	0.5418	0.8309	134.94	<0.0001

	Coefficient	Std. Err.	t	P>t	95% Conf.	Interval
Lnp**g**						
ln**g**_occupancy	−0.2411	0.0468	−5.16	<0.0001	−0.3334	−0.1489
ln**g**_bday_tbd	0.6620	0.0321	20.61	<0.0001	0.5986	0.7254
ln**g**_staff	0.2692	0.0301	8.94	<0.0001	0.2098	0.3287
Henan	−0.5654	0.1651	−3.42	<0.0001	−0.8912	−0.2397
constant	−1.3783	0.1917	−7.19	<0.0001	−1.7565	−1.0002
Lnp**m**						
ln**m**_occupancy	−0.0024	0.0613	−0.04	0.9690	−0.1236	0.1187
ln**m**_bday_tbd	0.7797	0.0349	22.32	<0.0001	0.7109	0.8488
ln**m**_staff	0.2755	0.0353	7.80	<0.0001	0.2058	0.3452
Henan	−0.4916	0.1514	−3.25	0.0010	−0.7904	−0.1928
constant	−0.8996	0.1958	−4.60	<0.0001	−1.2859	−0.5133
Lnp**s**						
ln**s**_occupancy	−0.1143	0.0287	−3.98	<0.0001	−0.1709	−0.0577
ln**s**_bday_tbd	0.7813	0.0289	27.05	<0.0001	0.7243	0.8383
ln**s**_staff	0.2914	0.0336	8.66	<0.0001	0.2250	0.3578
Henan	−0.4528	0.1469	−3.08	0.0020	−0.7427	−0.1629
constant	−1.1119	0.1883	−5.90	<0.0001	−1.4835	−0.7403
Lnp**p**						
ln**p**_occupancy	−0.3607	0.1310	−2.75	0.0060	−0.6191	−0.1023
ln**p**_bday_tbd	0.9024	0.0621	14.54	<0.0001	0.7799	1.0248
ln**p**_staff	0.3459	0.0795	4.35	<0.0001	0.1889	0.5027
Henan	−0.6970	0.2392	−2.91	0.0040	−1.1691	−0.2249
constant	−0.3166	0.2759	−1.15	0.2530	−0.8609	0.2277
Lnp**oth**						
ln**oth**_occupancy	−0.2559	0.0532	−4.81	<0.0001	−0.3609	−0.1509
ln**oth**_bday_tbd	0.6355	0.0356	17.86	<0.0001	0.5652	0.7057
ln**oth**_staff	0.2346	0.0559	4.19	<0.0001	0.1242	0.3449
Henan	−0.6787	0.1729	−3.93	<0.0001	−1.0197	−0.3376
constant	−1.6446	0.2572	−6.39	<0.0001	−2.1523	−1.1369

Observations = 41.

The signs of the estimated parameters are not surprising. The higher the number of staff and bed days in inpatient wards, the higher the ratio of their costs to that of costs incurred in the outpatient ward. The number of bed days consistently had the biggest proportional impact (e.g. the highest elasticity) on the ratio ranging from 0.9 percent points in the pediatrics ward to 0.66 percent points in the gynaecology ward. Given that the number of bed-days is included in the equation, the occupancy rate can be considered a measure of efficiency, so that holding bed-days constant, a higher occupancy rate reduces the ratio because of the increased efficiency.

As mentioned earlier, 40% of the data come from Henan district. The sign of the regression coefficient shows that the ratios were systematically lower in this province than in other parts of China, holding other explanators constant. Possible reasons for this are discussed subsequently.

As explained above, these results are not particularly interesting in themselves. They are interesting in that they allow us to estimate ward-specific average unit cost ratios, based on a set of defined characteristics. Table 3 shows the average unit cost ratios for the four main inpatient wards (the category “others” was not considered since it relates to such a wide range of wards that it is not particularly useful for policy purposes). These are based on the estimated proportions calculated from the model (see Eq. (5)) and the observed average values of the explanatory variables from the data set.

**Table 3:** Ratio of ward-specific unit cost per bed-day to the unit cost per outpatient visit.

Inpatient Wards	Mean	Std. Err.	[95% Conf. Interval]	
ratio_**go**	4.00	0.28	3.43	4.57
ratio_**mo**	4.64	0.33	3.98	5.31
ratio_**so**	4.12	0.29	3.53	4.70
ratio_**po**	3.14	0.24	5.33	7.45

Observations = 41.

Abbreviations: ratio_go = ratio of unit cost of Ob/Gyn to outpatient ward, mo = Internal medicine to outpatient, so = surgery to outpatient, po = pediatric to outpatient.

Because the average values of the explanatory variables mask the variation in the raw data, the average ward-specific unit cost per bed day varied between 3.14 to 4.64 times that of the unit cost per outpatient visit, much less than the variation observed in the raw data reported in Figure 1. All ward-specific unit cost ratios were significantly different to each other except the ratios of the unit cost per bed-day at the obstetrics/gynaecology and surgical wards (t test, p<0.0001).

To assess the level of accuracy and validity of the estimates derived from the model, Table 4 compares the ward-specific unit cost per bed day estimated from the model with the raw data, estimated for all but hospitals in the Province of Henan (discussed later). They were estimated as shown in Eq. (6) using the observed values of explanatory variables for each individual hospital in the data set. All estimates had good face validity and were not significantly different (t-test at 0.05 level of significance) from those developed using detailed step-down costing procedure (the raw data).

**Table 4:** Model estimates of ward-specific costs per bed-day in 2000 Chinese yuan (CY) excluding Henan Province (US$ in parenthesis: $1 = 8.3CY).

Variable	Raw data		Model		P value (t test)
	Mean	SD	Mean	SD	
uc**g**	282 ($34)	104 (13)	306 ($37)	143 (17)	0.24
uc**m**	327 ($39)	120 (14)	358 ($43)	179 (22)	0.09
uc**s**	287 ($35)	106 (13)	313 ($38)	154 (19)	0.13
uc**p**	229 ($28)	81 (10)	249 ($30)	114 (14)	0.20
uc**opv**	79 ($10)	28 (3)	72 ($9)	19 (2)	0.08

Observations = 24.

Abbreviations: uc = unit cost per bed-day, g = Ob/Gyn, m = internal medicine, s = surgery, p = pediatric, opv = outpatient visit.

## Discussion

In many countries there are little or no data on the costs of an inpatient stay in hospital, and even less on the costs of stays in a specific type of inpatient ward. Partly because the cost of undertaking full step-down costing studies is high, analysts seeking to assess the costs or cost-effectiveness of health interventions sometimes are forced to use the results of a single hospital costing study, or apply simple rules of thumb to allocate total hospital costs between inpatient care as a whole and the outpatient department [3]–[6]. In addition, general inpatient costs are sometimes used for an intervention requiring a particular type of inpatient care, regardless of whether the inpatient stay requires more or less care than other types of patients [5].

It has already been shown that analysts using the costs of general inpatient care taken from a single hospital run the risk of selecting an outlier that could be at least an order of magnitude different to the average costs of care in a particular type of hospital or setting [1], [3]. In this paper, we have extended this work to show four important additional results.

Firstly, the costs of specialized inpatient care can differ substantially depending on the ward. In our China case, the daily costs of a bed in one ward can be up to 17.4 times the cost of a bed in another ward, and up to 6.5 times the average cost of inpatient care. Analysts using the average costs of inpatient care in their studies run the risk of providing very misleading policy implications for interventions requiring specialty care.

Secondly, using average rules of thumb to allocate total hospital costs across specialty wards will also be inaccurate because of variability in key factors such as occupancy rates, the number of beds and staffing levels. More and better data is the solution, but in the meantime it would be possible to estimate the ratios of the costs per specialty ward to the cost of an inpatient visit in Chinese hospitals not covered by the data set, using the model described above. All that is required is information on occupancy rates, the number of beds and staffing level, as well as the total hospital cost. It remains to be seen if this could be replicated in other settings, or if a multi-country study could confirm the advantages of using a model to derive cost estimates for countries where data are not available.

Thirdly, the ratio of the unit cost of specialty wards to the unit cost of an outpatient visit also varies with the occupancy rate and staffing levels. This implies that it would be misleading to use a single unit cost per bed day to estimate the costs or cost-effectiveness of expanding coverage of interventions for example, something that is commonly done [3]–[6].

Finally, the results also reinforce the earlier suggestions that it is dangerous to base estimates of the costs or cost-effectiveness of interventions requiring hospitalization on data taken from a single hospital. This hospital might well be an outlier, biasing the results substantially.

We now return to the question of Henan province where the cost ratios were significantly lower than those estimated for the other 11 provinces in the data set. Consultation with the Henan Provincial Ward of Public Health clarified that during the period 1988 to 1998, the provincial government and its ministry of health had declared investment in the infrastructure (e.g., building) of public hospitals to be a priority. In China, public hospitals usually consist of two main buildings, one for outpatient services and one for inpatients. Each building includes general and specialty clinics and wards, but the outpatient building also includes the laboratory, radiology and imaging (e.g., Computerized Tomography, Magnetic Resonance Imaging) services. As a result, outpatient buildings in Henan absorbed most of the capital investment during this 10-year period. It is not surprising, therefore, to find lower cost ratios (due to higher costs of outpatient wards, the denominator of the ratio) there.

This paper offers an important methodological contribution to this field. The statistical basis for estimating department-specific cost-ratios used in our analysis has been enhanced by the adaptation of models for compositional data that were previously developed in other areas. These models take account of the key features of this type of data, namely that the fraction of costs attributable to each department is bounded by zero and one, and that all of the fractions must sum to unity. Smith and Kohn (2000) demonstrated that using a system of related equations such as the seemingly unrelated regressions lead to significant improvement in efficiency of the estimation through modelling, rather than ignoring, the correlated errors between equations [12].

Furthermore, the efficiency of the model has been increased by using department-specific information, which in addition to increasing the efficiency of the model, contributed to explaining a large proportion of the variability in costs between hospital departments [19]. The other practical advantage of using department-specific parameters in the model is that it allows analysts and hospital managers to vary the input parameters to tailor predictions to a particular setting, using different mixes of inputs and outputs.

While the work presented in this paper represents the first study to our knowledge of the extent and determinants of variation of hospital costs across wards further innovations may be possible in the future, including the development of compositional models designed specifically for panel data. It might also be possible to undertake a similar multi-country analysis to explain variation in determinants of ward-specific hospital costs across countries with a sufficiently large data set. If future applications confirm a standard relationship of the determinants of hospital costs across countries, or at least a predictable relationship allowing for varying hospital structures, this would be particularly valuable for planning and budgeting purposes especially for national scaling up estimates in countries where full step-down costing studies do not exist [23]–[25]. Although it would be preferable for analysts to estimate department-specific costs using the step-down procedure if time and financial resources permit, this paper shows that econometric analysis of existing data can provide useful estimates in the interim.

## Supporting Information

Appendix S1 **List of provinces included in the data-set.**
(DOCX)
