# Supplementary material for: Variability in Costs across Hospital Wards. A Study of Chinese Hospitals
Source: PLoS One. 2014 May 29;9(5):e97874. doi: 10.1371/journal.pone.0097874 (PMC4038551; doi:10.1371/journal.pone.0097874)
Supplement: Appendix S1 — List of provinces included in the data-set. (DOCX) [file pone.0097874.s001.docx]

Appendix S1. List of provinces included in the data-set

| Province | Frequency | Percent | Cumulative percent |
| --- | --- | --- | --- |
| Beijing | 1 | 2.44 | 2.44 |
| Fujian | 1 | 2.44 | 4.88 |
| Gansu | 1 | 2.44 | 7.32 |
| Guangdong | 3 | 7.32 | 14.63 |
| Guizhou | 2 | 4.88 | 19.51 |
| Hebei | 3 | 7.32 | 26.83 |
| Henan | 17 | 41.46 | 68.29 |
| Hubei | 3 | 7.32 | 75.61 |
| Jiangxi | 3 | 7.32 | 82.93 |
| Shandong | 1 | 2.44 | 85.37 |
| Sichuan | 2 | 4.88 | 90.24 |
| Zhejiang | 4 | 9.76 | 100.00 |
| Total | 41 | 100 |  |
